# NF-kappaB in Lung Tumorigenesis

**DOI:** 10.3390/cancers3044258

**Published:** 2011-12-14

**Authors:** Zhenjian Cai, Kam-Meng Tchou-Wong, William N. Rom

**Affiliations:** 1 Division of Pulmonary, Critical Care and Sleep Medicine, Department of Medicine, New York University School of Medicine, 462 First Avenue, NBV 7N24, New York, NY 10016, USA; E-Mails: zhenjian.cai@nyumc.org (Z.C.); kam-meng.tchou-wong@nyumc.org (K.-M.T.-W.); 2 Department of Environmental Medicine, New York University School of Medicine, 57 Old Forge Road, Tuxedo, NY 10987, USA

**Keywords:** NF-kappaB, lung cancer, tumorigenesis, tumor initiation, tumor promotion, tumor progression

## Abstract

The development of lung cancer in humans can be divided into three steps initiation, promotion and progression. This process is driven by alterations in related signal transduction pathways. These pathways signal the aberrant activation of NF-kappaB, a transcription factor that regulates the expression of genes important for lung tumorigenesis. Our current knowledge about the role of the NF-kappaB signaling pathway in the development of lung cancer has been bolstered by animal models demonstrating the connection between K-ras and tobacco induced lung transformation with NF-kappaB. Activation of downstream genes leads to cell proliferation, inhibition of apoptosis, angiogenesis, inflammation, invasion, and metastasis.

## 1. Introduction

Lung cancer remains the leading cause of cancer-associated deaths in the United States, despite the progress in targeted therapy of genetic subsets of lung cancer using tyrosine kinase inhibitors [1]. The development of lung cancer in humans is a complex process that can be divided into three steps: initiation, promotion and progression [2,3]. At the initiation step, mutations that inactivate tumor suppressor genes or activate oncogenes are established in the cell genome, which can induce transformation of mutant cells to acquire the ability to proliferate autonomously. Such “tumorigenic” mutations primarily are introduced into the genome by environmental irritants, although genetic predisposition of the host is also implicated in this process. For instance, tobacco smoke contains multiple carcinogens that can cause DNA damage in lung epithelial cells [4]. Ideally, as a fail-safe mechanism, the failure to repair damaged DNA should trigger cell death via apoptosis to maintain the genomic stability of the entire cell population. However, the signaling pathways regulating cell survival may be concomitantly activated during the cell injury and repair process, thereby increasing cell tolerance to damaged DNA. The increased survival of cells that fail to repair damaged DNA can lead to the accumulation of “initiated” cells harboring mutations in tumor suppressor genes and oncogenes essential for cell transformation. At the promotion step, tumors start to grow as a result of the accumulation of proliferative cancer cells. Tumor growth requires that mutant cells possess the ability to proliferate autonomously, a characteristic hallmark of cancer cells [5]. Hence, the signaling pathways regulating cell proliferation are usually constitutively activated during the process of tumor promotion. On the other hand, the microenvironment that consists of numerous cell types as well as non-cellular components such as the matrix surrounding the tumor also plays supportive roles for tumor growth by providing not only the “survival signal”, but also oxygen and nutrients [6,7]. At the progression step, the accumulated tumor cells acquire more aggressive phenotypes that further “differentiate” to undertake different “tasks”, notably, the facilitation of the tumor to invade surrounding tissues and to metastasize to remote organs. Underlying these behaviors of tumor cells are the aberrant alterations in the cell signaling pathways that control normal cell migration and adhesion. Growth factors, cytokines, and signaling cascades lead to transcription factor activation and migration into the nucleus where cancer-related genes are transcribed.

Nuclear factor-kappa B (NF-kappaB) represents a family of transcription factors that include five members in mammalian cells: p65 (RelA), RelB, c-Rel, p50/p105 (NF-kappaB1), and p52 (NF-kappaB2). These proteins bind DNA upon homo- or hetero-dimerization, which is mediated by the *N*-terminal Rel homology domain commonly shared among the family members. p65 (RelA), RelB and c-Rel all have a *C*-terminal transactivation domain and therefore can function as a transcription activator. p50/p105 (NF-kappaB1) and p52 (NF-kappaB2), on the contrary, lack the transactivation domain, and act as transcription suppressors [8]. By targeting a large number of genes involved in different signaling pathways, NF-kappaB regulates numerous distinct aspects of cellular physiology, such as inflammation, cell survival and proliferation.

Activation of NF-kappaB can be accomplished via three molecular pathways, namely the canonical pathway, the non-canonical pathway and the alternative pathway [9]. A common scheme shared by all three pathways is that: in unperturbed cells, NF-kappaB remains bound to the inhibitor of kappaB (I-kappaB) and thereby is sequestered in the cytoplasm. Upon activation by stimuli, I-kappaB is phosphorylated, which induces its ubiquitination and proteosome-mediated degradation. Subsequently, free NF-kappaB translocates into the nucleus to regulate transcription. The three pathways differ in that each pathway responds to a subset of extracellular and intracellular stimuli. Examples of stimuli that turn on the canonical pathway include DNA damage and cytokines such as TNF-alpha while the non-canonical pathway and the alternative pathway are activated under different conditions. Additionally, each pathway recruits different mediators for signal transduction. In the canonical pathway, I-kappaB kinase beta (IKK-beta) is the key mediator that phosphorylates I-kappaB, while in the non-canonical pathway, a paralogous kinase named IKK-alpha transduces the activation signal [8]. The alternative pathway, instead, employs a distinct kinase called casein kinase-2 (CK-2) rather than IKKs [10].

As a major signaling pathway controlling inflammation, cell survival and proliferation, abnormal activation of nuclear factor-kappa B (NF-kappaB) has been linked to the development of many cancer types, including lung cancer [9,11-13]. This review aims to summarize our current knowledge of the NF-kappaB signaling pathway underlying the process of lung tumorigenesis, focusing on the molecular mechanisms that lead to abnormal activation of NF-kappaB and the downstream molecular targets of NF-kappaB through which lung tumorigenesis initiates, promotes and progresses.

## 2. NF-kappaB and the Initiation of Lung Tumorigenesis

A committed step in the initiation of lung tumorigenesis is acquisition of tumorigenic mutations in lung epithelial cells. Several environmental factors are known to play important roles in this process, such as tobacco smoking and asbestos exposure. These environmental irritants contain multiple carcinogens that can cause DNA damage in lung epithelial cells via the generation of reactive oxygen species [14]. When cells fail to repair damaged DNA, they are subjected to programmed cell death to maintain the genomic integrity of the cell population. Ironically, however, these environmental risk factors, such as tobacco, can simultaneously induce chronic inflammation in lung tissue, which is accompanied by the activation of cell survival signaling pathways to promote cell survival.

As a major pathway in inflammation, NF-kappaB is activated during this process via the canonical pathway. Activated NF-kappaB can induce autocrine production of the inflammatory cytokine IL-6 and activation of the transcription factor STAT3, causing spontaneous lung cancer *in vivo* [15,16].

B-cell lymphoma 2 (Bcl-2), a critical inhibitor of apoptosis, is also an important target gene downstream of NF-kappaB. It has been shown that NF-kappaB inhibition can result in increased apoptosis of lung airway epithelial cells, which is associated with a marked reduction of Bcl-2 expression [17]. Thus, during tobacco exposure and chronic inflammation, elevated Bcl-2 expression exerts its anti-apoptotic effect and elevates the threshold for cell death, including those cells harboring damaged DNA. Consequently, the chances of developing a sporadic mutation in tumor suppressor genes or oncogenes are raised, leading to the initiation of lung tumorigenesis.

As lung tumorigenesis progresses, NF-kappaB activation is maintained primarily by tumor necrosis factor (TNF) that induces activation of IKK beta via the canonical pathway. Activation of NF-kappaB serves as a survival signal that contributes substantially to the resistance to TNF-induced cell death [18-20].

## 3. NF-kappaB and the Promotion of Lung Tumorigenesis

### 3.1. NF-kappaB in K-ras Induced Lung Transformation

Mutations of numerous genes have been linked to lung tumorigenesis, with K-ras being a well-established prototype [21]. K-ras is one of the first oncogenes discovered more than 30 years ago. As a crucial player in many signaling networks, K-ras connects a variety of upstream signals to an even wider set of downstream pathways that functions in balancing cell survival, growth, apoptosis and senescence. Thus activating mutations of K-ras result in cell transformation and uncontrolled cell proliferation. The significance of K-ras mutations in lung tumorigenesis can be highlighted by the facts that K-ras mutations are found in up to about 30–50% of lung adenocarcinomas, and that lung cancers harboring K-ras mutation are more aggressive with worse prognosis [22].

Recent studies in various cells lines and mouse models showed that NF-kappaB is required for K-ras induced lung transformation. Using low-passage primary immortalized human small airway cells and their K-ras-transformed counterparts, Basseres *et al.* [23] showed that the NF-kappaB activity is upregulated in K-ras-transformed cells. Similar results were obtained in a mouse model of K-ras-induced lung cancer. Furthermore, deletion of the NF-kappaB subunit p65/RelA reduces the number of K-ras induced lung tumors and tumors with p65/RelA deletion have higher numbers of apoptotic cells, reduced spread and lower tumor grade. These results highlighted the important roles of NF-kappaB in K-ras induced lung tumorigenesis.

In K-ras transformed lung epithelial cells, NF-kappaB is activated via the canonical pathway. It has been observed that in lung cancer cell lines carrying oncogenic K-ras mutation, NF-kappaB activation is dependent on the kinase activity of IKK beta, a key regulator in the canonical pathway of NF-kappaB activation [20]. However, it remains largely unclear how K-ras transduces the activation signal to IKK beta, although studies in embryonic fibroblasts suggest that this process requires the signaling adaptor p62 [24]. Notably, NF-kappaB activity is down-regulated by the tumor suppressor p53. In both primary mouse embryonic fibroblasts and lung tumor cells lines expressing oncogenic K-ras (G12D) and lacking p53, restoration of p53 inhibits NF-kappaB signaling [25].

Once activated in the lung epithelial cells, NF-kappaB promotes cell proliferation at least in part by regulating the transcription of two downstream target genes, namely, induction of Cyclin D1 and suppression of Phosphatase and tensin homolog (PTEN). Cyclin D1 is a cell cycle regulator that drives cells to enter the S phase. It has been demonstrated that upon exposure to 4-(methylnitrosamino)-1-(3-pyridyl)-1-butanone (NNK), a tobacco-specific carcinogen, NF-kappaB is activated that subsequently up-regulates the protein level of Cyclin D1 and promotes cell proliferation in normal human bronchial epithelial cells and small airway epithelial cells [26].

PTEN functions as a negative regulator of phosphatidylinositol-3 kinase (PI3K)/Akt-mediated cell survival pathway. Thus, PTEN is a tumor suppressor gene which is mutated in many hereditary and sporadic human cancers. Since NF-kappaB activates the transcription of Snail, a transcription suppressor of PTEN, PTEN is negatively controlled by NF-kappaB [27]. It has been demonstrated in a subset of human lung cancer cells that NF-kappaB activation was necessary and sufficient for inhibition of PTEN expression. It is worthwhile to point out that there is a second mechanism for PTEN suppression, which is independent of the DNA binding and transcription activity of NF-kappaB. The NF-kappaB subunit p65, but not p50, sequesters the limiting pools of CBP/p300, a transcriptional coactivator, thus down-regulating the expression of PTEN. As a result, the PI3K/Akt pathway is released from inhibition, which promotes cell proliferation and survival [28].

### 3.2. NF-kappaB in Tobacco-Induced Promotion of Lung Tumorigenesis

In addition to its role as tumor initiator, tobacco smoke also acts as a promoter for lung tumorigenesis. It has been shown that repetitive exposure to tobacco smoke promotes tumor development in carcinogen-treated mice and in transgenic mice undergoing K-ras activation in lung epithelial cells. This tumor-promoting effect is dependent on the activation of NF-kappaB pathway in myeloid cells since ablation of IKK beta activity in myeloid cells inhibits proliferation of transformed lung epithelial cells [29]. However, it remains to be defined how myeloid cells communicate with lung epithelial cells to stimulate their proliferation.

### 3.3. NF-kappaB in Tumor Angiogenesis

Angiogenesis is the formation of new blood vessels that supply nutrients and oxygen necessary for the proliferation of tumor cells. Angiogenesis is considered one of the hallmarks for tumor growth and increased angiogenesis is related to poor prognosis [30].

Angiogenesis is mediated by a number of growth factors and cytokines, including basic fibroblast-like growth factor (bFGF), vascular endothelial growth factor (VEGF), angiopoietin 2 (Ang2), interleukin 8 (IL8) and transforming growth factor beta (TGF-beta), all of which promote the proliferation of endothelial cells [31]. Hypoxia-inducible factor (HIF) is a major group of transcription factors that upregulate the transcription of these growth factors and thereby stimulating angiogenesis in response to hypoxia [32].

Recent studies identified cross-talks between the NF-kappaB and HIF signaling pathways. The promoter region of HIF-alpha contains a canonical NF-kappaB binding site and mutation of this site resulted in the loss of hypoxia-induced HIF-alpha expression [33]. Indeed, further study found that NF-kappaB upregulates HIF-alpha protein level both *in vivo* and *in vitro* [34]. Interestingly, it has also been found that HIF-alpha promotes NF-kappaB signaling by up-regulation of p65 and IKK-alpha in neutrophils [35]. In addition, NF-kappaB plays an important role in HIF-independent angiogenesis. Under hypoxic condition, NF-kappaB can be activated in IKK-beta dependent manner as a result of down-regulation of prolyl hydroxylase [36]. Once activated, NF-kappaB in turn activates the transcription of VEGF, IL-8, Ang-2, and other factors necessary for angiogenesis [37-39]. Malignant progression of normal alveolar epithelial cells to adenocarcinoma in K-ras mice was associated with enhanced intralesional vascularity and neutrophilic inflammation characteristic of exaggerated IL-8 function; neutralizing antibody to IL-8 abrogated these effects in the K-ras mouse model [38]. *In vitro* studies demonstrate IL-8 involvement in cell growth and migration, particularly in NSCLC cell lines with K-ras mutations, and siRNAs against K-ras downregulate IL-8 [39].

## 4. NF-kappaB and the Progression of Lung Cancer

Two characteristic features of cancer cells are their invasive and metastatic abilities. Normal cells are orderly arranged via a number of proteins that mediate interactions and attachment of cells to each other and to extracellular matrix (ECM). Abnormalities of these proteins account for the loss of architecture and gain of invasive ability of cancer cells. Metastasis refers to the process whereby cancer cells invade the lymph-vascular structure, migrate to and continue growing at distant sites within the body. Cancer invasion and metastasis are distinct but continuous processes and therefore they are discussed together here.

Cancer invasion and metastasis are multi-step processes including detachment from neighboring cells, attachment to and digestion of extracellular matrix, migration through the damaged matrix, and adherence to new sites within the body. Recent studies suggest that NF-kappaB is also involved in tumor invasion and metastasis by regulating the transcription of several target genes.

### 4.1. NF-kappaB and CRMP-1

NF-kappaB promotes tumor invasion by down-regulating expression of collapsin response mediator protein-1 (CRMP-1), an invasion suppressor gene. CRMP-1 encodes an intracellular phosphoprotein and was found to be suppressed or lost in several human cancers, especially primary malignant brain tumors and lung cancers. In lung adenocarcinoma Cl1-5 cells, which have high metastatic potential, CRMP-1 expression was demonstrated to be greatly suppressed and overexpression of CRMP-1 inhibited their metastatic ability, confirming the critical role of CRMP-1 in the invasion of lung cancer [40]. Subsequent studies showed that NF-kappaB controls CRMP-1 expression. The NF-kappaB p50 protein, which is constitutively expressed and strongly induced by TNF-alpha in Cl1-5 cells, binds directly to the kappaB site in the CRMP-1 promotor region and suppresses CRMP-1 expression. This interaction plays critical roles in regulating the invasive behavior of C11-5 cells since transfection of antisense p50 gene into Cl1-5 cells increased the CRMP-1 protein level and decreased the invasive activity of Cl1-5 cells [41].

### 4.2. NF-kappaB and Lung Cancer Metastasis

The fact that NF-kappaB is important for lung cancer metastasis has been proven by several studies using various animal models. Staphopoulos *et al.* [42] used a model of intravenous injection of Lewis lung carcinoma cells into immunocompetent C57Bl/6 mice, and they found that induction of lung inflammation by NF-kappaB activation in airway epithelial cells potentiates the metastasis of lung carcinoma cells. We have previously shown that expression of I-kappaB beta, which binds to NF-kappaB and sequesters it in the cytoplasm, in lung adenocarcinoma A549 cells significantly inhibited metastasis when injected intravenously via tail vein of nude mice [43]. In an experimental murine cancer metastasis model in which a colon adenocarcinoma cell line generates lung metastases, injection of bacterial lipopolysaccharide (LPS) stimulated tumor growth in lung tissue, which was dependent on both TNF-alpha production by host hematopoietic cells and NF-kappaB activation in tumor cells. Furthermore, inhibition of NF-kappaB in both colon and mammary carcinoma cells converted the LPS-induced growth response to LPS-induced tumor regression [44].

NF-kappaB exerts its metastasis-promoting effect by regulating the transcription of a subset of genes known to be important for tumor metastasis, such as prometastatic matrix metalloproteinase 9 (MMP9), a urokinase-like plasminogen activator (uPA), which is MMP9 activator, and heparanase. MMP9 and heparanase are degradation enzymes with functions of breaking down extracellular matrix, facilitating cell migration and invasion of lymph-vascular structures. In a murine lung alveolar carcinoma cell line, NF-kappaB blockade results in the down-regulation of MMP9, uPA, and heparanase, as well as the reciprocal up-regulation of antimetastatic tissue inhibitors of matrix metalloproteinases 1 and 2 and plasminogen activator inhibitor 2. The resulting effect of NF-kappaB blockade is the prevention of intravasation of tumor cells in an *in vivo* chick chorioallantoic membrane model of metastasis as well as inhibition of metastasis in a murine model [45]. On the contrary, it has been reported that in lung cancer PC-14 cells, NF-kappaB inhibition suppresses expression of KAI1/CD82, a known metastasis suppressor for several human cancer types [46]. The latter may be related to the p53 status since the PC-14 cells utilized in this study are p53-deficient.

In addition, NF-kappaB can promote tumor metastasis through activation of the alphavbeta3 (avb3) integrin. Integrins are transmembrane proteins that link extracellular matrix to intracellular cytoskeletons and signaling molecules and play important roles in cellular migration, adhesion, growth and differentiation. Expression of integrin is up-regulated in most cancers and studies have shown that integrin promotes tumor metastasis not only via promoting adhesion of tumor cells at distant metastatic sites but also by activating cellular proliferating pathways. One of the extensively studied pathways for metastasis is the CCL-5-mediated metastasis pathway. CCL-5 is a member of the CC-chemokine family that has been shown to be important for cancer migration and metastasis. It has been shown that NF-kappaB is activated in the CCL-5-mediated lung cancer cell migration and metastasis pathway. In this pathway, CCL-5 activates the PI3K and Akt signaling pathway, which in turn activates NF-kappaB. NF-kappaB then leads to the induction of expression of avb3 integrin which contributes to the migration of human lung cancer cells [47]. Interestingly, another study has shown that avb3 integrin can activate the NF-kappaB pathway. In this study, Fong *et al.* [48] found that osteopontin, which is abundant in bone matrix and functions in cell adhesion, migration and proliferation, when bound to its receptor, avb3 integrin, can activate the PI3K/Akt/NF-kappaB/avb3 integrin pathway. Collectively, these studies support the existence of a positive feedback pathway between avb3 integrin and NF-kappaB, which functions to promote cellular proliferation and tumor metastasis.

## 5. Conclusions

In this review, we have summarized that aberrant activation of NF-kappaB in lung epithelial cells and interestingly in myeloid cells mediates initiation, promotion and progression of lung tumorigenesis. It is worth to point out here that NF-kappaB may play even more roles than mentioned above in the development of lung cancer, as exemplified by two most recent studies. In a first study, it is demonstrated that lung cancer cells bearing an activating mutation of epithermal growth factor receptor (EGFR) were sensitized to apoptosis by concurrent treatment of inhibitors targeting the NF-kappaB signaling pathway [49]. This suggests that NF-kappaB is clearly involved in the EFGR-induced lung tumorigenesis, although NF-kappaB only seems to be one of the several pathways that mediate the tumorigenic effect of the activating EGFR mutation. A second most recent study revealed that constitutive NF-kappaB activation in alveolar epithelial cells stimulates the recruitment of regulatory T lymphocytes, inducing the formation of tumor by compromising cell immunity [50]. This result further links chronic inflammation to lung tumorigenesis.

The significance of NF-kappaB activation in lung tumorigenesis suggests that inhibition of this signaling pathway provides novel strategies for the prevention and treatment of lung cancer. Indeed, many synthetic chemical compounds that target the NF-kappaB pathway are being tested for cancer therapeutic effect both *in vivo* and *in vitro* with promising outcomes [51]. It is hopeful that as we increase our understanding of the regulation of the NF-kappaB pathways, insights into the better design of drugs that effectively target NF-kappaB will be gained that will ultimately lead to better prevention and treatment of this devastating disease.
